# Instability of retroviral vectors with HIV-1-specific RT aptamers due to cryptic splice sites in the U6 promoter

**DOI:** 10.1186/1742-6405-4-24

**Published:** 2007-10-17

**Authors:** Stephen E Braun, Xuanling Shi, Gang Qiu, Fay Eng Wong, Pheroze J Joshi, Vinayaka R Prasad, R Paul Johnson

**Affiliations:** 1 Division of Immunology, New England Primate Research Center, Harvard Medical School, One Pine Hill Drive, Southborough, MA 01772 USA; 2 Albert Einstein College of Medicine, Bronx, 1300 Morris Park Avenue (Room GB 401), Bronx, NY 10461 USA; 3 J. David Gladstone Institute of Virology and Immunology, University of California at San Francisco, 1650 Owens Street, San Francisco, CA 94158 USA; 4 Partners AIDS Research Unit and Infectious Disease Unit, Massachusetts General Hospital, Boston, MA 02115 USA

## Abstract

**Background:**

Internal polymerase III promoters in retroviral vectors have been used extensively to express short RNA sequences, such as ribozymes, RNA aptamers or short interfering RNA inhibitors, in various positions and orientations. However, the stability of these promoters in the reverse orientation has not been rigorously evaluated.

**Results:**

A series of retroviral vectors was generated carrying the U6+1 promoter with 3 different HIV-1 RT-specific RNA aptamers and one control aptamer, all in the reverse orientation. After shuttle packaging, the CD4^+ ^cell line CEMx174 was transduced with each vector, selected for expression of GFP, and challenged with HIV-1. We did not observe inhibition of HIV-1 replication in these transduced populations. PCR amplification of the U6+1 promoter-RNA aptamer inhibitor cassette from transduced CEMx174 cells and RT-PCR amplification from transfected Phoenix (amphotropic) packaging cells showed two distinct products: a full-length product of the expected size as well as a truncated product. The sequence of the full-length PCR product was identical to the predicted amplicon sequence. However, sequencing of the truncated product revealed a 139 bp deletion in the U6 promoter. This deletion decreased transcriptional activity of the U6 promoter. Analysis of the deleted sequences from the U6 promoter in the antisense direction indicated consensus splice donor, splice acceptor and branch point sequences.

**Conclusion:**

The existence of a cryptic splice site in the U6 promoter when expressed in a retroviral vector in the reverse orientation generates deletions during packaging and may limit the utility of this promoter for expression of small RNA inhibitors.

## Background

The tRNAs, U6 small nuclear (sn) RNA and adenovirus-virus-associated RNAs are normally expressed in cells at high levels by RNA polymerase III. These polymerase III promoters have been used in gene therapy applications to express a variety of inhibitory RNAs, including RNAi, aptamers, ribozymes, antisense RNAs, and decoy RNAs [1-5]. Retroviral and lentiviral vectors have been the primary method of gene delivery to carry these inhibitor cassettes [1,4]. Importantly, high levels of expression and inhibitory activity have been demonstrated in several studies targeting HIV-1 sequences with RNA polymerase III-driven inhibitory RNAs [4,5].

While stable transduction of the transgene and vector sequences is necessary for gene expression, several studies reporting lack of gene function after transduction with retroviral vectors have identified various mechanisms limiting vector function. In several systems including our studies, the expression cassettes have been positioned in the vectors the in the reverse orientation to allow for the inclusion of important regulatory sequences (poly A signals, introns, self-splicing ribozymes, etc.) so that these signals will not affect vector genomic RNA production. However, deletions or aberrant processing have been identified in several of these studies due to the introduction of new cryptic regulatory sequences. For β-globin expression, which is normally developmentally tissue-specific regulated, generation of a retroviral vector with the β-globin gene in the reverse orientation lead to the identification of several RNA termination signals in the insert [6]. Jonsson *et al *also found fortuitous splice sites in the purine nucleoside phosphorylase (PNP) genomic construct when inserted in a retroviral vector in the reverse orientation [7]. Additionally, multidrug resistance 1 (MDR1) expression in transduced bone marrow and spleen colonies resulted in both truncated and full-length mRNA, because the cDNA was found to contain cryptic splice donor and splice acceptor sites, even though it is expressed in the forward direction [8]. Additionally, when a retroviral vector contains direct repeats, deletions can occur during reverse transcription with RNA template misalignment of the polymerase growing point and the first direct repeat [7,9]. It has been suggested that regulatory sequences may potentially emerge in retroviral vectors from sequences that are not naturally transcribed (i.e. antisense sequences or the U6 promoter) [10].

Using retroviral vectors to introduce HIV-1 specific RNA aptamers into CD4^+ ^cell lines, we found consensus splicing signals in the reverse orientation of the U6 promoter that lead to partial deletion of the U6 promoter after retroviral-mediated gene transfer. The splicing deletion lead to reduced promoter activity, and low expression of the aptamer in transduced cells was associated with a lack of HIV-1 inhibition. These studies could potentially be generalized to other vector systems that express small RNA inhibitors from this pol III promoter and should serve as a warning to other investigators in the design of their gene delivery vectors.

## Results

### Transduction of CEMx174 cells with the retroviral vectors

**Figure 1:**
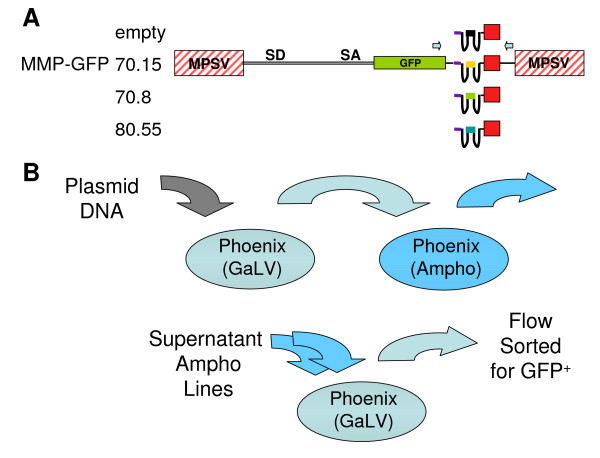
**Diagrams of the retroviral vectors and the shuttle-packaging scheme**. A) The MMP-based vector series contains GFP transcriptionally regulated by the MPSV LTR and the control or 3 different HIV-1 specific RT aptamers transcriptionally regulated by the U6+1 promoter positioned in the antisense orientation. The forward and reverse arrows above GFP and the 3' UTR represent the PCR primers used to amplify the inhibitor cassette in transduced cells. B) Generation of high-titer producer cell lines by shuttle packaging. Vector plasmids were transiently transfected into Phoenix (GaLV) packaging cell line and supernatant was used to transduce Phoenix (amphotropic) packaging cell lines. After selecting for Phoenix (amphotropic) cells expressing GFP, supernatants were collected for titering and to transduce Phoenix (GaLV) packaging cell lines with 2 exposures. Supernatants from these Phoenix (GaLV) cells were collected for titration and to transduce the CD4^+ ^cell line CEMx174.

Previous studies have demonstrated effective inhibition of HIV and SHIV-RT viral replication in aptamer-transduced cells [11]. Towards evaluating the *in vivo *efficacy of the HIV-1 RT-specific RNA aptamers, we sought to generate high-titer stable Phoenix(amphotropic) [12] and Phoenix(GaLV) [13] packaging cell lines. Therefore, we shuttled packaged the 4 MMP-GFP aptamer vectors (Figure 1a) [11] by transient transfection through Phoenix(GaLV) packaging cell lines and stable transduction of Phoenix(amphotropic) packaging cell lines (Figure 1b). Expression of GFP in the empty-, 70.15-, 70.8-, and 80.55-transduced Phoenix(amphotropic) cells averaged 14% before sorting (Table 1) and >95% after sorting. When used for packaging of other MMP-GFP vectors, the titers of the Phoenix(GaLV) cells were consistently less than 1 × 10^5 ^IU/ml (data not shown). Therefore, Phoenix(GaLV) cells were transduced with two serial exposures to supernatant from the sorted Phoenix(amphotropic) cells. Expression of GFP in the empty-, 70.15-, 70.8-, and 80.55-transduced Phoenix(GaLV) cells averaged 65% before sorting (Table 1) and >95% after sorting. Molecular analysis of the packaging cell lines after sorting (Table 1) indicated that the Phoenix(amphotropic) cells had <1 copy per cell (0.82, 0.33, 0.80, and 0.97 copies, respectively), while the Phoenix(GaLV)-empty, -70.15, -70.8, and -80.55 cells had on average 26 copies of integrated provirus per cell (29, 26, 15, and 33 copies, respectively).

**Table 1:** Characterization of Phoenix (Amphotropic) and Phoenix (GaLV) packaging cell lines.

**MMP Vector**	**Phoenix (Amphotropic)**			**Phoenix (GaLV)**		
	Percent GFP^1^	Proviral copies^2^	Titer (TU/ml)^3^	Percent GFP^1^	Proviral copies^2^	Titer (TU/ml)^3^

Empty	7%	0.82	1.0 × 10^5^	68%	29	2.6 × 10^5^
70.15	18%	0.33	1.4 × 10^5^	69%	26	2.5 × 10^5^
70.8	17%	0.80	1.1 × 10^5^	59%	15	2.1 × 10^5^
80.55	14%	0.97	1.2 × 10^5^	64%	33	2.5 × 10^5^

^1^Initial percentage of GFP^+ ^cells in each packaging cell line after transduction and prior to cell sorting.

^2^Molecular analysis of number of proviral copies of the GFP gene in each packaging cell line as determined by real-time PCR of genomic DNA.

^3^Calculated titer of the supernatant from each packaging cell line after limiting dilution and analysis of the expression of GFP in U2OS cells.

To determine the number of infectious particles generated by each cell line, supernatants from these producer cell lines were used to transduce the human osteosarcoma cell line U2OS, and GFP expression was evaluated in the U2OS cells 48 hours post-transduction. As shown in Table 1, the titer of the amphotropic supernatants ranged from 1.1 to 1.4 × 10^5 ^infection units per ml (IU/ml), and the titer of the GaLV-pseudotyped viruses ranged from 2.1 to 2.6 × 10^5 ^IU/ml. These stable high-titer producer cells were used to generate both GaLV-pseudotyped and amphotropic viral particles.

**Figure 2:**
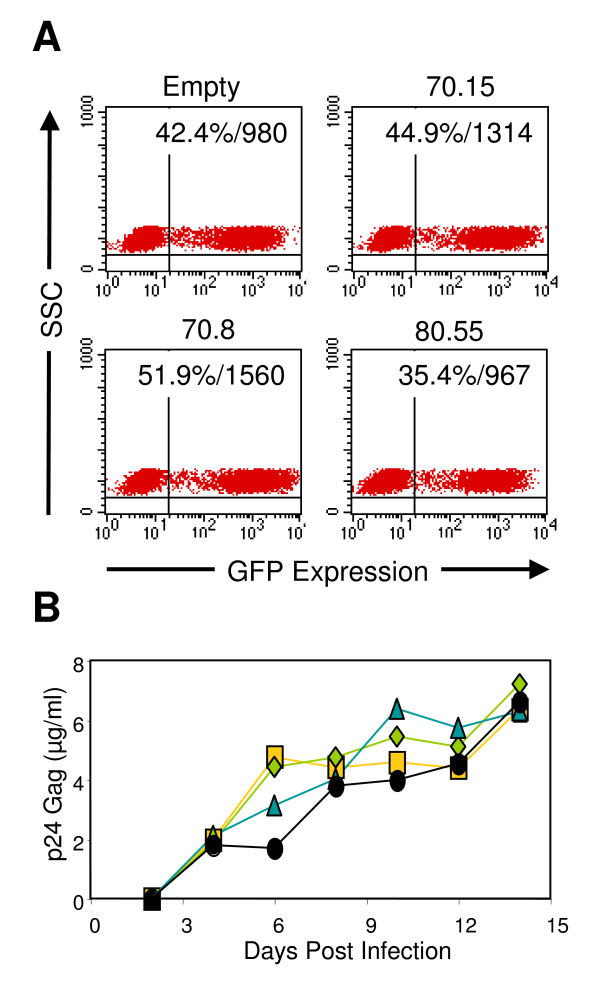
**Inhibition of HIV-1_NL4-3 _in aptamer transduced CEMx174 cells**. A) Transduction of the CD4^+ ^cell line CEMx174. CEMx174 cells were transduced with each MMP-aptamer vector by spinoculation and overnight incubation with Phoenix (GaLV) supernatants. Cells were washed and expanded for 48 hours before analysis by flow cytometry. The dot plots indicate GFP expression versus side scatter characteristics for the empty vector (left plot), 70.15 vector (left center), 70.8 vector (right center), and 80.55 vector (right plot) before sorting. The percentage of GFP^+ ^cells and the mean fluorescent intensity are indicated in the upper right corner of the plot. B) Challenge of aptamer-transduced CEMx174 cells with HIV-1_NL4-3_. Aptamer-transduced CEMx174 cells (empty, filled circle; 70.15, yellow square; 70.8, green diamond; 80.55, blue triangle) were sorted for expression of GFP, infected with HIV-1_NL4-3 _(MOI = 0.01 TCID_50 _per cell) and followed over 14 days for production of p24 Gag by ELISA.

To evaluate the efficacy of these vectors to inhibit HIV-1 replication, we transduced CEMx174 cells, a CD4^+ ^cell line, with the GaLV-pseudotyped aptamer vectors (Figure 2a). The gene transfer efficiency of the empty, 70.15, 70.8, and 80.55 aptamer into the CEMx174 cells was 42%, 45%, 52%, and 35%, respectively. Expression of GFP, as determined by flow cytometry, was strong with mean fluorescent intensities of 980, 1314, 1560, and 967 units. After sorting for expression of GFP, aptamer-transduced CEMx174 cells were challenged with HIV-1_NL4-3 _(MOI = 0.01 TCID_50_/cell, Figure 2b and MOI = 0.001 TCID_50_/cell, data not shown) and assessed by ELISA for gag production in the supernatant for two weeks. In contrast to previous results that used a single round of packaging and pools of individual CD4^+ ^clones after limiting dilution plating [11], we did not find any significant inhibition of viral replication in the heterogeneous populations of aptamer-transduced CEMx174 cells (Figure 2b).

### Mechanism for the lack of inhibition in aptamer-transduced CEMx174 cells

**Figure 3:**
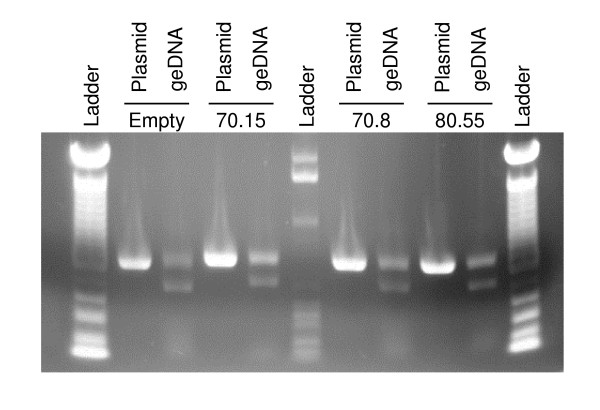
**Instability of the aptamer cassette in transduced CEMx174 cells**. Genomic DNA from transduced cells and plasmid DNA from the different vectors were PCR amplified with a forward primer from GFP (3'GFP-for) and a reverse primer from the untranslated region of the vector (3' UTR-rev). PCR reactions were separated by gel electrophoresis and stained with ethidium bromide. The expected size of the MMP-70.15 amplicon is 654 bps; the expected size for the other bands varies depending on the length of the aptamer. The DNA ladders are either the 100 bp DNA ladder or the 1 kb DNA ladder from NEB.

To explain the lack of inhibition we observed in our aptamer-transduced CEMx174 cells, we hypothesized that the retroviral vector was rearranged during shuttle packaging and transduction. Since we had sorted the transduced CEMx174 cells for expression of GFP, any structural rearrangements affecting the inhibitor cassette would need to be downstream of the GFP sequences. Therefore, we designed PCR primers to specifically amplify the aptamer inhibitor cassette in the 3' UTR of MMP-GFP (Figure 1a). PCR amplification of the four-retroviral plasmids generated the correct sized bands (Figure 3), excluding the possibility of a PCR artifact. However, PCR amplification of genomic DNA from all four populations of aptamer-transduced CEMx174 cells generated two bands on the agarose gel (Figure 3) – one full-length product and another truncated product approximately 100–150 bps smaller than the full-length product.

**Figure 4:**
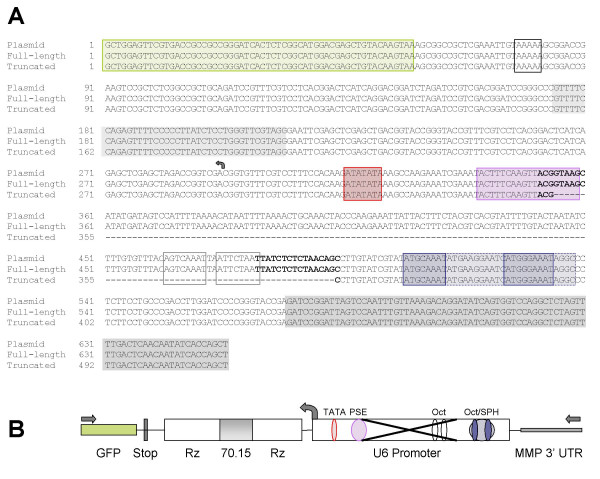
**Sequencing analysis of the truncated PCR product**. A) Multiple sequence alignment of the full-length and the truncated PCR products from 70.15-transduced CEMx174 cells with the PCR product from plasmid template. The sequence of the full-length clones match the 654 bps predicted sequence from the plasmid template. The truncated product aligns with the predicted sequence except for a 139 bp deletion. The colored boxes indicate the transcription factor binding sequences: TATA box (red), the PSE (plum), and the sequences related to the octamer consensus (ATTTGCAT, blue), the protected SPH domain (light blue), while the grey boxes are non-functional octamer-related sequences [19, 20, 28]. The grey shading is the sequence of the HIV-1-specific RT aptamer 70.15 and the black box is the pol III termination signal. The bold sequences are the proposed splice donor and splice acceptor sequences. B) Schematic map of the predicted amplicon in forward orientation as described in Figure 1. The black arrows indicate the amplification primers. The boxes represent from left to right the 3' end of GFP, the Pol III termination sequence, a self-splicing ribozyme, the HIV-1-specific RT aptamer 70.15 (grey box), a self-splicing ribozyme, the human U6+1 promoter, and MMP 3' UTR sequences. The line represents linker sequences between the functional sequences. The spotted ovals represent the TATA box (red) and PSE (plum) in proximal promoter; the non-function octamer sequences (grey), the Staf-binding domain (light blue) and the two octamer-related sequences (blue) in the distal promoter all within the U6 promoter. A curved arrow represents the position and direction of the transcriptional start site. The black X represents the splicing deletion.

We initially examined whether the deletion was caused by recombination of the short repeat elements in the flanking ribozyme domains because of misalignment during reverse transcription. To determine the nature of the deletion, we gel purified and cloned the truncated product, the full-length product, and the plasmid product for sequencing analysis. Individual clones were isolated and sequenced. The consensus sequences of the clones from the full-length PCR products and from the plasmid amplicon are identical to the predicted amplicon sequence (Figure 4a). In contrast, the sequence alignments from 20 of 21 clones from the truncated PCR product contain an identical 139 bp deletion in the predicated amplicon sequence (Figure 4a), while one clone contained the full-length sequence. When the deletion was compared to the predicted amplicon sequence, the deletion was mapped from bases -62 to -200 (relative to the transcriptional start site) in the middle of the U6 promoter (Figure 4a). These data demonstrate that the deletion in the inhibitor cassette did not occur in the sequences between the repeat elements in the self-splicing ribozymes but occurred in the promoter region during post-transcriptional processing of the mRNA, potentially from splicing of retroviral genomic mRNA.

**Figure 5:**
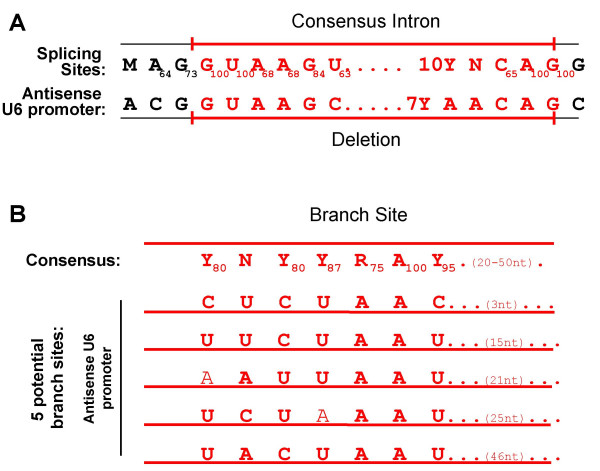
**Comparison of the antisense U6 promoter with splicing consensus sequences**. A) Top row: the consensus splice donor and splice acceptor sequences with the intron sequences (red bold with a red bold line above) and the exon sequences (normal font with a thin line above). The necessity for the internal pyrmidine stretch is represented by the 10Y. The nucleotide-usage percentage at each position is noted as a subscript. M equals adenosine or cytosine, Y equals pyrimidines and R equals purine. Bottom row: the antisense U6 promoter sequences surrounding the 139 bp deletion with the sequences that remain (in normal font with a thin line below) and the sequences that were deleted (in red bold with a red bold line below). B) Top row: The consensus sequences for the internal branch point are represented with the nucleotide-usage percentage noted as a subscript. The distance of the invariant adenosine to the splice acceptor site is noted in parentheses. Potential branch sites: 5 potential branch sites in the antisense U6 promoter are indicated. Divergent sequences are shown in normal font and conserved sequences are shown in bold. All sequences are intronic and shown in red. The distances to the end of the deletion are noted in parentheses.

To address whether inadvertent RNA splicing of the inhibitor cassette in the antisense orientation was responsible for generating the deletion, we compared the antisense sequence in the deletion of the U6 promoter to consensus splicing signals. As shown in Figure 5a, the GU and AG at the ends of most introns, along with the internal stretch of pyrimidines and other frequently occurring nucleotides, define the consensus sequences for splice donor and splice acceptor sites (reviewed in [14-16]). In most exons, the unspliced sequence is (A/C)AG at the donor site and G at the acceptor sites (Figure 5a) [14-16]. However, the sequences at the intron/exon junction are not sufficient to signal the presence of an intron. The branch point is another required sequence usually located 20 – 50 bases upstream of the acceptor [14-16]. The consensus sequence of the branch point is "CU(Pu)A(Py)", where adenosine is absolutely required for splicing (Figure 5b) [14-16]. Additional positive and negative recognition elements are also necessary [14-16]. Interestingly, the junctions of the deleted sequences in the U6 promoter contain the requisite GU and AG sequences and show very strong similarities to the splice donor splice acceptor consensus sequences with an internal pyrimidine stretch (Figure 5a). Because the branch point consensus sequences are more flexible, the antisense U6 promoter has potential branch sites 3, 15, 21, 25, and 46 bps upstream of the splice acceptor site (Figure 5b). All these sites contain the requisite adenosine. The few divergent sequences in these potential branch points are shown in normal font.

**Figure 6:**
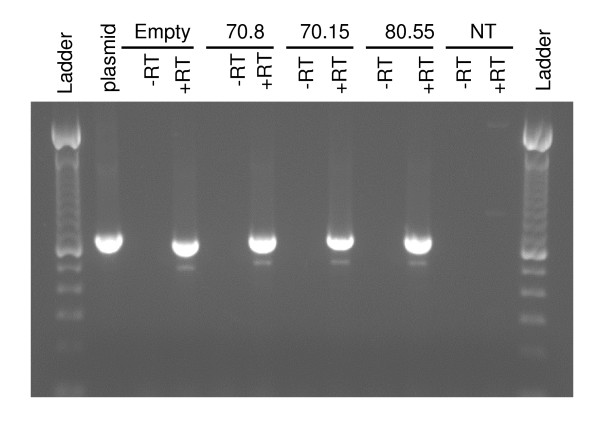
**Deletion of aptamer cassette in RNA transcripts from the Phoenix(amphotropic) packaging cell lines**. The Phoenix (amphotropic) packaging cell lines were transfected with the different aptamer vectors. Total RNA was isolated and subjected to amplification by RT-PCR with (+RT) or without (-RT) reverse transcriptase using the 3'GFP-for and 3' UTR-rev primers. Amplification of the MMP 70.15 plasmid was included as control. The DNA ladder is the 100 bp DNA ladder from Invitrogen.

To determine whether the truncated aptamer cassette originated during post-transcriptional splicing of the mRNA or after later steps in the retroviral life cycle, we isolated total RNA from the Phoenix (amphotropic) packaging cell lines after transfection with each of the aptamer vectors. Under these conditions, the inhibitor cassette will have been transcribed and the RNA processed, but will not have progressed to reverse transcription and/or integration. The total RNA from the packaging cell line was amplified by RT-PCR with or without reverse transcriptase using the same primer combination (Figure 1a) as was used previously to amplify the aptamer in Figure 3. In all four transfected Phoenix (amphotropic) populations, both the full-length and the truncated product were detected (Figure 6), while amplification of the MMP-70.15 plasmid generated only the full-length product and amplification of the RNA samples without RT were negative (Figure 6). The ratio of full-length to truncated product during one round of transcription and mRNA processing favors the full-length product (Figure 6). Because the RT-PCR reaction was not designed or evaluated for quantitative analysis, the efficiency of RNA processing cannot be determined. These results demonstrate that the deletion in the U6 promoter when transduced in the antisense orientation within a retroviral vector is caused by splicing of the retroviral genomic RNA at cryptic splice donor/splice acceptor sequences.

**Figure 7:**
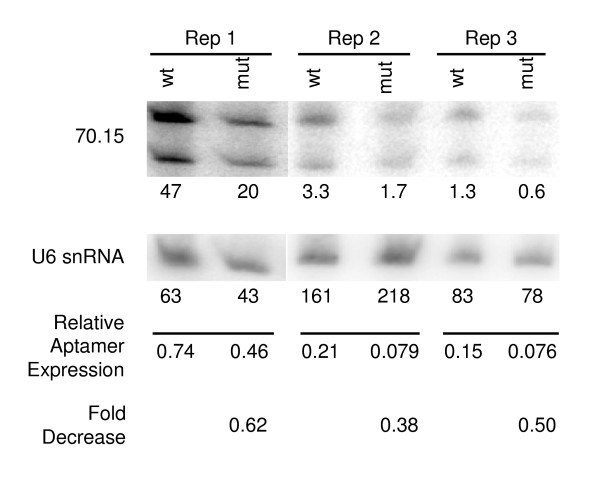
**Northern blot of wildtype and mutant U6 promoter activity**. Plasmid DNA with the wildtype (wt) and mutant U6 promoters transcriptionally regulating the aptamer 70.15 were transfected into 293T cells. After 48 hours, total RNA was isolated, separated by electrophoresis, blotted and probed for expression of the aptamer 70.15 and of the U6 snRNA (endogenous control). Shown are 3 replicates for the aptamer and the U6 snRNA. The intensity of each band (x10^4 ^units) was quantified with the STORM 820 phosphorimager and is shown below the image. A relative ratio of the aptamer 70.15 signal to the endogenous U6 snRNA expression is shown. The fold decrease of mutant U6 promoter activity and Wt U6 promoter activity in regulating transcription of the aptamer 70.15 is indicated below the line for each replicate. The reduction in expression of the mutant U6 promoter was 0.50 ± 0.12 fold (mean ± standard deviation [SD]).

Comparisons of the deletion with the functional domains of the U6 promoter revealed that the deletion removes 5 of 19 bps in the proximal sequence element (PSE) and two non-functional Oct-1 binding domains in the distal enhancer, but not the functional Oct-1 and SPH elements in the distal enhancer (Figure 4). The 139 bp deletion also changes the spacing between the enhancer element and the PSE within the U6 promoter to only 13 bps (Figure 4). Previous studies have shown that the promoter is transcriptionally active with limited distance between the PSE and the upstream enhancer elements [17]. To determine whether the deletion had an effect on the transcriptional activity of the U6 promoter, we used the pCRII plasmids containing full-length and truncated U6 promoter-70.15 amplicons to transiently transfect 239T cells and quantified expression of the 70.15 aptamer by Northern blot (Figure 7). Endogenous expression of the U6 snRNA gene was used to normalize samples (Figure 7). The mutation in the U6 promoter consistently reduced expression of the 70.15 aptamer compared to the wildtype U6 promoter (0.50 ± 0.12, mean ± SD) but did not completely eliminate expression (Figure 7). Thus, deletion of 139 bps within the U6 promoter, either through loss of a portion of the PSE and/or through alterations in the spatial arrangement of transcription factors, lowered U6 promoter activity.

## Discussion

Towards a stem cell gene therapy strategy for AIDS, we studied transduction of three different HIV-1-specific RNA aptamers and one control aptamer transcriptionally regulated by the U6 promoter in the antisense orientation within a retroviral vector. After shuttle packaging and transduction of CD4^+ ^cell lines, these vectors were no longer able to inhibit HIV-1 replication. Instead, we found deletions within the U6 promoter in all 4 vectors after retroviral mediated gene transfer and consensus splicing signals in the U6 promoter in the reverse orientation.

The classical splicing consensus sequences and basal splicing machinery have been described for many years [14-16]. The basal splicing machinery recognizes the classical splicing sequences and catalyzes the reaction removing the intron and joining the exons together. At the ends of the exon and introns are the well-described splice donor-splice acceptor sequences (see Figure 5) and within the intron is an internal branch point with a requisite adenosine that is necessary for the intramolecular lariat bond. Additionally, auxiliary elements within exon or introns are commonly required for efficient constitutive or alternative splicing. Finding similar classical splicing consensus sequences surrounding the deletion in the U6 promoter suggests that the splicing of this cryptic intron during transcription of the retroviral genomic RNA in the packaging cell lines caused the deletion.

Although the antisense U6 promoter closely matches the consensus splice donor/splice acceptor sites and has 5 potential internal branch sites, splicing of the antisense U6 promoter is inefficient. In the RT-PCR analysis of aptamer expression in cells transfected with the four different aptamer vectors, we observed only a small fraction of the shorter deleted product after only a single cycle of transcription and potential RNA splicing. In the genomic DNA from transduced CEMx174 cells after the retroviral vector was shuttle packaged, amplification of both the full-length and truncated products was observed, even after the vector underwent multiple rounds of viral replication (i.e., transcription, reverse transcription and integration). It appears that the mismatches in the splicing consensus sequences or that other auxiliary splicing signals may be necessary for efficient splicing of the antisense U6 promoter. Although splicing is detectable, the frequency is low, and this provides some optimism for vectors that have been produced with the U6 promoter in the antisense direction. However, our finding a similar deletion in four separate MMP-aptamer transduced populations reinforces that this splicing-induced deletion occurs reproducibly and that multiple cycles of retroviral packaging and transduction would only serve to increase the fraction of truncated product.

The molecular mechanism of transcriptional activation with U6 promoters has been thoroughly studied [18]. RNA polymerase (pol) II promoters and pol III promoters for snRNA genes are very similar, even using common binding factors (reviewed in [18]). The U6 pol III promoter contains 3 protein-binding domains: the proximal sequence element (PSE) and the TATA box are approximately 50 and 25 bps upstream of the transcriptional initiation site and the distal enhancer element is approximately 200 bps upstream of the transcriptional initiation site. For basal transcription, the PSE binds the snRNA activating protein complex (SNAPc) of five proteins (as they do in the pol II PSEs), while the TATA box binds to the initiation complex TFIIIB (reviewed in [18]). TFIIIB, like the TFIIB for pol II promoters, facilitates interactions with the RNA polymerase and is required for all pol III promoters, even the separated tRNA-type promoters. For the human U6 promoter, TFIIIB consists of the TATA box binding protein (TBP), TFIIIB50 and TFIIIB90. Meanwhile, the distal enhancer element binds multiple factors (Oct-1, ZNF143 and ZNF76) that stimulate the formation of preinitiation complexes, with some proteins activating both pol II and pol III snRNA gene transcription. Correct nucleosome positioning between the enhancer and PSE during chromatin assembly also brings into close proximity the two binding domains and allows for cooperative interactions with the Oct-1 and SNAPc. A stable initiation complex recruits either RNA pol II or III and results in snRNA transcription.

The effects of the splicing deletion we observed in the aptamer vectors on cooperative binding and transcriptional activity may be complex because the 139 bps splicing deletion mutates potential transcription factor binding sites and changes the alignment of the remaining sites. In the proximal promoter, five of 19 bps from the PSE are deleted, which may limit SNAPc binding [18]. In the distal U6 enhancer, two of the 4 total octamer consensus sequences (ATTTGCAT [19]) are also deleted. We do not believe loss of these two non-functional [19] domains contributes to the decreased promoter activity, since the deleted U6 promoter still contains the two functional octamer binding sequences: 1) the SPH motif, which binds to ZNF76 and ZNF143 [20], and 2) the Oct-1 binding sites, which is involved in the cooperative binding of SNAPc to the U6 promoter that leads to increase recruitment of pol III and transcriptional initiation [21]. Additionally, the splicing deletion may affect transcriptional activity because correct nucleosome positioning in the U6 promoter allows for the juxtaposed positioning of the distal enhancer with the proximal PSE necessary for their cooperative interactions [22]. Thus, removing 134 bps from the U6 promoter will change the spatial alignment of the transcription factors with only 13 bps remain between the two DNA binding domains. In gel shift assays, cooperative binding of Oct-1 and SNAPc still occurs in recombinant promoters with only 18, 23, and 27 bps separation [17]. Our Northern blots demonstrate that the deletion in the U6 promoter decreased, but did not completely eliminate, transcriptional activity. Some limited interaction must be possible between the SNAPc on the PSE and with Oct-1 in the enhancer.

Many factors in the configuration of retroviral vectors play a role in gene expression and activity. We initiated these studies when we observed a lack of inhibition of HIV-1 replication in cells that had been transduced with HIV-specific aptamer vectors generated using a shuttle packaging protocol. Interestingly, we found the splicing deletion in the U6 promoter that reduced transcriptional activity. However, not all transduced cells contained the splicing deletion, indicating that the transduced populations contained a mixture of wildtype and deleted promoters. In our earlier work [11], we showed inhibition of HIV-1 with a gamma-retroviral vector expressing GFP and containing the U6-aptamer cassettes in the reverse orientation. These previous studies used a single round of packaging and pools of individual clones after limiting dilution plating for expression and challenge studies. In the present studies, we shuttle packaged the same vectors between amphotropic- and GaLV-pseudotyped Phoenix packaging cell lines to generate higher titer clones for subsequent *in vivo *experiments. In the process, amplification of the vector during shuttle packaging also allowed for several cycles of RNA processing and the enrichment of aptamer cassettes with the splicing deletion in transduced cells. Additionally, the present studies used bulk populations of transduced cells (sorted for GFP expression) rather than pools of clones. The position-dependent effects of the integration site in the T cell clones may have resulted in higher levels of aptamer expression than we observed in bulk populations of transduced cells with heterogeneous integration sites. Consequentially, abundant aptamer expression was detected previously in the T cell clones by RNase protection assay [11], while aptamer expression in the bulk transduced cells analyzed in this report was not detected by northern blot and RNase protection assay (data not shown). While the splicing deletion we observed in the U6 promoter reduced transcriptional activity, other factors in the vector design must also contribute to the overall lack of aptamer expression and viral inhibition that we observed in the transduced cells. We are currently examining other pol III promoters in the reverse and forward orientations to determine which vectors will permit stable gene transfer, the highest level of expression and the strongest inhibition. Interestingly, many gene transfer studies with the U6 promoter have used the complete U6 promoter, even though a minimal promoter with only the distal and proximal elements is active. Recently, the U6 promoter has also been used in lentiviral-based retroviral vectors to regulate siRNA inhibitors of either HIV-1 or of host cellular genes [4,23,24]. These SIN-type lentiviral vectors, with a deletion in the 3' LTR to inactivate the HIV-1 promoter after reverse transcription and integration, have the U6 promoter-inhibitor cassette in the forward direction, and thus would not be subject to the deletion via the cryptic splice site described in this report.

## Conclusion

We found deletions of the U6 promoter that matched closely with the classical splicing consensus sequences when transferred in a retroviral vector in the antisense orientation. The deletion reduced the transcriptional activity, and the lack of detectable aptamer expression in bulk transduced cells was associated with a lack of HIV-1 inhibition. These studies could potentially be generalized to other vector systems that express small RNA inhibitors from this polymerase III promoter and should serve as a warning to other investigators in the design of their inhibitor vectors.

## Methods

### Cell culture and transduction conditions

The CD4^+ ^cell line CEMx174 was cultured in RPMI-1640 (Sigma, St. Louis, MO) plus 20% fetal bovine serum (FBS, HyClone, Logan, UT), 10 mM HEPES (Cellgro/Mediatech, Fisher Scientific, Federal Way, WA), 50 U/ml penicillin and 50 μg/ml streptomycin (Cellgro/Mediatech), 2 mM L-glutamine (Cellgro/Mediatech), (R20 medium) at 37°C with 5% CO_2_. CEMx174 cells were exposed to viral supernatants plus 8 μg/ml polybrene (Sigma) with a 20 minutes spin at 200 rpm and then overnight culture. After 24 hours, the cells were washed and expanded in R20 media. Phoenix (amphotropic) [12], Phoenix (GaLV) packaging cell lines [13] (both based on 293T cells), and U2OS cells were cultured in DMEM plus 10% FBS, 10 mM HEPES, 50 U/ml penicillin and 50 μg/ml streptomycin, 2 mM L-glutamine (D10 medium) at 37°C with 5% CO_2_.

### Generation and evaluation of high-titer producer cell lines

The MMP vectors were previously described [11]. Plasmid DNAs of the four retroviral vectors was transfected into the Phoenix (GaLV) packaging cell line by calcium phosphate co-precipitation and washed after 24 hours. After 48 hours, culture supernatant was collected, passed through a 0.45-micron filter and used to transduce the Phoenix (amphotropic) packaging cell line. Aptamer-transduced Phoenix (amphotropic) cells were sorted for expression of GFP using a Becton Dickinson FACS Vantage. These heterogeneous populations of cells were used to generate supernatant for titering and for transducing Phoenix (GaLV) packaging cell lines. Aptamer-transduced Phoenix (GaLV) cells were sorted for expression of GFP. These heterogeneous populations of cells were used to generate supernatant for titering and for transducing CEMx174 cells. Dilutions of viral stocks were used to transduce U2OS cells and the percent GFP positive cells determined by flow cytometry. Stock titers ranged from 1.0 to 2.6 × 10^5 ^transduction units (TU)/ml (Table 1).

### Viral inhibition assays

The HIV-1 strain NL4-3 was kindly provided by Ronald C. Desrosiers (NEPRC, HMS). Viral stocks were generated by infection of CEMx174 cells and harvesting cell-free supernatants on day 8–12 after infection. Virus stocks were analyzed for Gag production by ELISA (HIV p24, Coulter HIV-1 Core Antigen Assay; Coulter International Corp., Miami, FL) per the manufacturer's instructions and/or titered for TCID_50 _values by limiting dilution assay as previously described [25].

To challenge aptamer-transduced and control CEMx174 cells for viral replication, transduced cells were resuspended for 4 hours in HIV-1 at MOIs of 0.001 to 0.01 TCID_50_/cell, before being washed and cultured in 15 ml R20. Production of p24 Gag in the supernatant was assessed by ELISA (Beckman Coulter, Fullerton CA) in cell-free supernatants to quantify viral replication.

### Molecular analysis

Genomic DNA was isolated from transduced cells using the QIAamp Blood Mini Kits (Qiagen, Valencia, CA). The inhibitor sequence from each transduced population was amplified using the forward 3'GFP (5'-GCT GGA GTT CGT GAC CGC-3') and the reverse MMP 3' UTR (5'-AGC TGG TGA TAT TGT TGA GTC AAA AC-3') primers as shown in Figure 1. Reaction mixtures containing 500 ng genomic DNA, 200 nM of each primer, 0.2 mM dNTPs in 50 μl of 1× PCR buffer (Invitrogen Corp., Carlsbad, California) were amplified with annealing temperature of 56°C, elongation temperature of 72°C, and denaturation temperature of 94°C for 35 cycles, using Platinum Taq DNA polymerase (Invitrogen). Samples were separated by agarose electrophoresis and stained with ethidium bromide. The image was captured using the Alpha Innoteck Corp. Imager program version 3.24 (Alpha Innoteck Corp., San Leandro, CA).

The PCR product for each band was gel purified (Qiagen) and cloned into pCR^®^II-blunt-TOPO^® ^using Zero Blunt TOPO PCR Cloning Kit (Invitrogen). At least 18 individual clones for each PCR product were isolated and sequenced on a CEQ™8000 Genetic Analysis System (Beckman Coulter, Fullerton CA) using CEQ™DTCS-Quick Start Kit (Beckman Coulter) according to the manufacturer's instructions.

Sequence data and chromatogram data from the individual clones were aligned using ContigExpress in the Vector NTI Suite (Invitrogen). Consensus sequences were generated and compared to the predicted amplicon sequence using BEAUTY (BLAST Enhanced Alignment Utility) [26], an enhanced version of the Basic Local Alignment Search Tool (BLAST) from the National Center for Biotechnology Information (NCBI), on the Baylor College of Medicine website [29]. Multiple sequence alignments were generated using the Multiple Sequence Alignment Program [27] at the Baylor College of Medicine website.

The Phoenix (amphotropic) packaging cell line was transfected with the different retroviral plasmids containing the HIV-1-specific RT aptamers using lipofectamine 2000 (Invitrogen) 48 hours prior to total RNA isolation with using Qiagen RNA easy (Qiagen). The inhibitor sequence from each transfected population was amplified using the same forward 3'GFP and the reverse MMP 3' UTR primers. The reaction mixtures contained 1 μg of total RNA and 200 nM of each primer, 0.2 mM dNTPs, 1.6 mM MgSO_4_, in 50 μl of 1× One-step Master mix (Invitrogen). The samples was reverse transcribed at 56°C for 30 mins with (RT^+^) or without (RT^-^) 25 units of the reverse transcriptase Superscript III (Invitrogen), and amplified with annealing temperature of 56°C, elongation temperature of 72°C, and denaturation temperature of 94°C for 40 cycles, using Platinum Taq DNA polymerase (Invitrogen). Samples were separated on 1.2% agarose gel and stained with ethidium bromide. The image was captured using the Alpha Innoteck Corp. Imager program version 3.24 (Alpha Innoteck Corp., San Leandro, CA).

### Northern Blot

The pCRII TOPO plasmids corresponding to the wildtype (wt) and splicing mutant (mut) U6 promoter-70.15 aptamer expression cassettes were transiently transfected into 293T cells with lipofectamine 2000 (Invitrogen) per the manufacturer's instructions. As a control for the transfection efficiency, the constitutively active SEAP expression cassette was also transfected and soluble SEAP activity quantified. Two days after transfection, total RNA was isolated from the aptamer-expressing 293T cells by Trizol (Invitrogen). The amount of RNA in each sample was calculated from the optical density at 260 nm (OD_260_). RNA samples were subjected to electrophoresis in 8% denaturing polyacrylamide gel, transferred to a nylon membrane (Hybond N+, Amersham Pharmacia Biotech) and hybridized with a ^32^P-labeled RNA aptamer, tRNALys3 or U6 snRNA probe. After stringent washing of the membrane, images were captured and the bands were quantified by the phosphorimager (STORM 820; Molecular Dynamics).

## Abbreviations

GaLV gibbon ape leukemia virus

LTR long-terminal repeat

MDR1 multidrug resistance 1

mut mutant

Oct-1 octamer transcription factor-1

PNP purine nucleoside phosphorylase

pol RNA polymerase

PSE proximal sequence element

RT reverse transcriptase

SEAP secreted embryonic alkaline phosphate

SIN self-inactivating

SPH SphI postoctamer homology element

SNAPc snRNA activating protein complex

snRNA small nuclear RNA

Staf selenocysteine tRNA gene transcription activating factor

TBP TATA box binding protein

TCID_50 _50% tissue culture infectious dose

TU transduction units

UTR untranslated region

wt wildtype

ZnFs zinc fingers DNA bind protein

ZNF143 human ortholog of *Xenopus *Staf

ZNF76 human ortholog of *Xenopus *Staf

## Competing interests

The author(s) declare that they have no competing interests.

## Authors' contributions

PPJ participated in the design of and created the retroviral vectors. FEW carried out the viral production (packaging, titering and transductions) and viral challenge experiments (culture and immunoassays). GQ carried out the viral challenge experiments (culture and immunoassays), the molecular genetic studies and participated in the sequence alignments. XS performed the Northern blots and quantified expression. VRP participated in the design of the vectors and the subsequent study. RPJ participated in the design of the vectors and the subsequent study, and edited the manuscript. SEB participated in the design of the vectors and the subsequent study, coordinated the study, performed the sequence alignment, matched the consensus sequences and wrote the manuscript. All authors read and approved the final manuscript.
